# Fabrication of a low-cost, small-footprint modular lab-scale wet spinning system

**DOI:** 10.1016/j.ohx.2026.e00752

**Published:** 2026-02-28

**Authors:** Joseph A. Houghton, Richard S. Blackburn

**Affiliations:** Leeds Institute of Textiles and Colour, School of Design, University of Leeds, LS2 9JT, UK

**Keywords:** Textiles, Wet-spinning, Modular, Lab-scale, Fibres

## Abstract

Textile production is one of the largest industries on the planet. Global annual fibre production was over 113 million tonnes (Mt) in 2021 and is predicted to increase to 149 Mt by 2030. Research into this area has potential to have huge economic and environmental impacts, however the equipment needed to perform this research is often prohibitively expensive and only available on large scale, with quoted ‘lab-scale’ equipment still taking up the footprint of a small laboratory and costing hundreds of thousands of pounds.

This paper details the design and fabrication of a truly lab-scale, modular wet spinning system for rapid first principle research into wet spun fibres. The total fabrication costs for this system are in the region of £500–700 and the total footprint required is <1 m^2^. The system also packs down for easy transportation and storage and requires a singles mains plug socket to operate.

This system has already been used to explore a variety of wet spun fibres including cellulosics, alginates and caseins and has been used to develop new, novel dyeing systems for wet spun fibres as published in ASC Sustainable Chemistry and Engineering: https://doi.org/10.1021/acssuschemeng.3c07437.

## Graphical abstract

**Figure:**
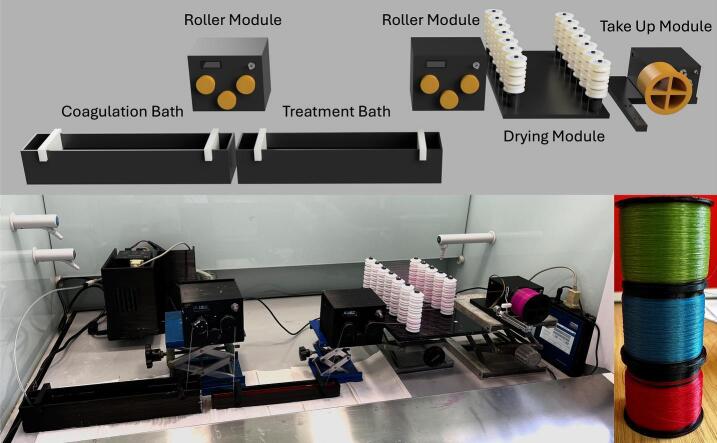

Specifications table

**Table:** 

Hardware name	The Incy Wincy Wet Spinning Rig (IWWSR)
Subject area	Engineering and materials science Chemistry and biochemistry Educational tools and open source alternatives to existing infrastructure General
Hardware type	Mechanical engineering and materials science
Closest commercial analog	No commercial analog is available at this scale, however at pilot scale there are many systems such as the FET-200 series, the Anytester AT235 or the Areka Wet Spinner.
Open source license	CC BY 4.0
Cost of hardware	*∼£500-700*
Source file repository	https://doi.org/10.17632/776w7sj2f2.1

## 1 Hardware in context

Wet spinning is the process of dissolving a polymer in a suitable solvent to make a viscous spin dope, then pumping the dope through a narrow aperture or spinneret into a coagulation anti-solvent where the polymers solidify into fibres through exchange of the dissolution solvent with the coagulation solvent *via* diffusion. This formed fibre can then be treated, washed, drawn (stretched) and dried to affect its eventual properties. Common fibres that utilise wet spinning in their production include viscose, acrylic, lycra among others [1]. The equipment used for this process is relatively simple in nature, but available systems are either very large industrial scale (1 tonne of fibre produced per hour) or pilot-scale R&D systems (∼1 kg of fibre per hour). There is currently no available example of a truly lab-scale wet spinning system capable of producing gram quantities of fibre easily, reproducibly and without excessive cost, space requirements and chemical usage/waste.

A prime example of a current small scale wet spinning system is that produced by Fibre Extrusion Technology (FET) Leeds UK [2]. Their standard small-scale wet spinning system has a 5 x 2 m footprint, a coagulation bath with a volume of 65 L and a cost of £200,000 for the basic, un-customised model; any modification, for example bath coatings to allow for acidic media, would incur extra costs. This cost, footprint and limitations are broadly similar across any commercially available small-scale wet spinning system, such as the Anytester AT235 Bench-Top Wet Spinning Machine [3] (5 x 1 m) and the Areka Wet Spinner [4] (5 x 0.8 m). These systems are not designed for the same purpose as the system described herein, and the lab-scale, modular wet spinning system described in this paper does not replace systems or services provided by companies such as FET. It merely allows for low-risk, low volume and rapid experimentation of wet spinning processes before taking it forward to small-pilot scale using one of these commercially available systems, which is a vital step in preparation for tonne scale industrial application.

Wet spinning is an area of high research potential with hundreds of research papers devoted to advances in the field being published every year. The challenge comes from the reproducibility of the work performed in these papers. Often an R&D lab will either make use of one of the large-scale, commercially available systems described previously or, more commonly, fabricate their own system in-house such as Nechyporchuk et al. [5], Cui et al. [6] or Chen et al. [7]. While this allows for research to be performed in the host organisation, it limits the potential for collaborative research outside as these systems are not readily available or reproducible. Often little to no information on the exact spinning system is included in the publication (as the novelty is the use or the treatment of the fibre, *etc*.) but this means that any researcher wanting to replicate/expand upon the research performed will have to firstly develop their own spin system from scratch, and then repeat all the work performed to ensure that the mechanical system does not detrimentally impact the chemistry of the process.

This paper describes a low-cost, simple to fabricate wet spinning system that requires only a 3D printer and a soldering iron to produce, using off the shelf components and having a footprint of < 1 x 1 m.

## 2 Hardware description

As previously mentioned, the expenditure, footprint and know how required to run commercially available wet spinning systems is a large hurdle preventing more exploratory research into wet-spinning and wet-spun fibres. The hardware described herein looks to alleviate many of these barriers by providing a cheap, simple to fabricate and fix, and easy to use, truly lab scale wet spinning system.

### 2.1 Cost

From the beginning of the design for this system, the aim was to make it as low-cost as possible. This was because the Author had identified that there was little to no cost-effective systems for early-stage exploration of wet spinning research. The current iteration of the system costs between £500-750 depending on design choices. This is roughly 0.25% the cost of the closest commercial analogue. It is important to note that this is sans pump. Pumps capable of dealing with the viscosities involved in wet spinning can be expensive, but for initial experiments a simple syringe pump performs adequately (there are limitations in the viscosity of the dope and the speed of spinning, but for initial trials these are minor issues). Syringe pumps can be purchased for as little as around £1000; alternatively there are papers detailing the fabrication of syringe pumps in HardwareX [8], [9].

The full system is not required for wet spinning, as each module is designed to work independently, therefore the capital expenditure (cap-ex) of the system can be spread over time as the research develops. Initially a pump, coagulation bath and roller are all that are required to produce lengths of fibre, which represents a cost of around £100. Modules can then be added as and when needed over time. The cost for replacement/fixing the system has also been kept as low as possible, with each part being modular, and the electronics in each module being easy to swap for a new part if needed. The most expensive part of each module is the PCB board with all components soldered on: if this fails it represents a cost of roughly £70-80 to replace depending on the configuration – this is the maximum amount of money that would be needed to fix a module unless more than one part simultaneously broke.

### 2.2 Ease of fabrication

Having the equipment, facilities and know how to fabricate equipment is not something that every company/institution has. Therefore, it was decided that when designing this system, only the bare minimum of equipment and knowledge should be required, to allow for more people to successfully build and use it. The only equipment required are a 3D printer (parts have been tested on an Ender 3 Neo Pro, an Ultimaker 2+ and a Bambu Lab X1) and a standard soldering iron. The circuitry has been designed to be simple and intuitive, to make the fabrication process as easy as possible, PCBs have been designed with labels and diagrams for the componentry and header pins/wires to the periphery components. As working with high voltages and currents is dangerous and should only be conducted by trained personnel, 12 V and 1 A is the maximum voltage and current this system is designed to use, which can be obtained by a standard UK mains plug adaptor. The code required to run the Arduinos is completely open source, and has been fully commented for aid in understanding. However, no coding experience is needed to run the system and the only time a change is needed in the code is if modifications to the system are needed (for example: change the roller/spool diameter, swap out to a different motor, *etc*.).

### 2.3 Ease of use

Another barrier to the use of the currently available wet spinning systems, is the complexity of their operation, usually requiring training by the manufacturing company and usually means there is a handful of skilled operators that know how to use the system safely – increasing risk of losing this knowledge, especially in academic institutions where research staff turnover is usually high. This system alleviates this issue by having each module separately controlled (no need for a centralised control panel or PC to run) and control is achieved by a single potentiometer (for the standard roller modules) and two potentiometers and a button (for the final take up module with optional rasterization module). Because each module is independently controlled, this enhances the modularity of the system, allowing individual parts to be used in isolation if the entire system is not required. Due to the limitations of plug sockets in standard fume hoods, a power block adaptor was designed so that all modules could be controlled from a single, mains plug socket, this allows other electrical equipment to be used (pump, drying fan etc.) while also running up to 6 modules simultaneously.

### 2.4 Solvent/chemical waste

For exploratory R&D, material use should be kept to a minimum to reduce wastage (as described in the 12 principles of green chemistry [10]. Existing wet spinning systems commonly require upwards of 0.5–1 kg of spin dope and in excess of 65 L of coagulation solvent. This is a large volume of solvent and polymer to commit to a process that is early in its development cycle. The system described herein can be run using a standard syringe pump (and therefore require 1–10 mL quantities of spin dope) and the coagulation bath as standard has a 500 mL volume (although smaller baths can be used) representing a 50x reduction in polymer required and 13x reduction in coagulation solvent required, meaning far less waste generated for early trials.

### 2.5 Size Requirements

As covered previously, the smallest commercially available wet spinning rig has a footprint of 5 x 2 m, this can be reduced by clever positioning to allow for vertical stacking of the different processes, but this brings further complications involving working at height. For a standard laboratory space, a 5 x 2 m footprint is just too large for a single piece of equipment, and if hazardous or volatile solvents are to be used there is the extra consideration of fume extraction and safety. Ideally a truly lab-scale R&D wet spinning system would fit inside a standard chemistry fume extractor cupboard. These range in size depending on the institution but are generally at least 1 x 1 m. The system described herein fits comfortably inside a 1 x 1 m fume hood and is easy to break down and pack away (its packed-down dimensions are roughly 0.4 x 0.4 m x 0.3 m meaning that experiments can be set up, run and packed away in a standard workday (essential where fume hood space is shared between multiple researchers/groups).

### 2.6 Portability

The fact that the system packs down into a relatively small volume also opens the potential for travel with the system. Instead of having partners making lab visits to see the research being performed, the system can instead be transported for showcases. This also opens the potential for wet spinning to be showcased at conferences, expos and even taken into schools to help educate the next generation of textile scientists. This system allows researchers:

• Low-cost and low-risk first-principle research into wet spun fibres, with cap-ex of <£1000 and need for very little solvent and chemicals.
• Rapid throughput of wet spinning experiments, with fast set up and running time for the system and the ability to spin km of fibre within a day.
• Truly lab-scale wet spinning with rig footprint being <1 m^2^ allowing for use inside a fume hood if hazardous materials are being used. Packs down rapidly into an even smaller footprint, beneficial for shared laboratory spaces.
• Due to the simplistic design choices, allows for rapid training and use by new users, including for educational purposes.
• Allows users to spin novel wet-spun fibres at a scale that allows for initial textile exploration (e.g. knit and weave) and could potentially be used to create small amounts of high value fibre (up to around 5 km per day).

## 3 Design files summary

Table 1 shows a summary of all design files for this system.

**Table 1:** Design files summary.

Design file name	File type	Open source license	Location of the file
Bath 58 mm.stl	STL File	CC BY 4.0	https://doi.org/10.17632/776w7sj2f2.1
Bath 58 mm.step	CAD file	CC BY 4.0	https://doi.org/10.17632/776w7sj2f2.1
Bath 50 mm.stl	STL File	CC BY 4.0	http://doi.org/10.17632/776w7sj2f2.1
Bath 50 mm.step	CAD file	CC BY 4.0	http://doi.org/10.17632/776w7sj2f2.1
Bath 29 mm.stl	STL File	CC BY 4.0	http://doi.org/10.17632/776w7sj2f2.1
Bath 29 mm.step	CAD file	CC BY 4.0	http://doi.org/10.17632/776w7sj2f2.1
Coagulation Bath 50 mm Suba_Seal.stl	STL File	CC BY 4.0	http://doi.org/10.17632/776w7sj2f2.1
Coagulation Bath 50 mm Suba_Seal.step	CAD file	CC BY 4.0	http://doi.org/10.17632/776w7sj2f2.1
Fibre Clip 58 mm.stl	STL File	CC BY 4.0	https://doi.org/10.17632/776w7sj2f2.1
Fibre Clip 58 mm.step	CAD file	CC BY 4.0	http://doi.org/10.17632/776w7sj2f2.1
Fibre Clip 50 mm.stl	STL File	CC BY 4.0	http://doi.org/10.17632/776w7sj2f2.1
Fibre Clip 50 mm.step	CAD file	CC BY 4.0	http://doi.org/10.17632/776w7sj2f2.1
Fibre Clip 29 mm.stl	STL File	CC BY 4.0	http://doi.org/10.17632/776w7sj2f2.1
Fibre Clip 29 mm.step	CAD file	CC BY 4.0	http://doi.org/10.17632/776w7sj2f2.1
Spinneret Clip 50 mm.stl	STL File	CC BY 4.0	http://doi.org/10.17632/776w7sj2f2.1
Spinneret Clip 50 mm.step	CAD file	CC BY 4.0	http://doi.org/10.17632/776w7sj2f2.1
Roller Module Case.stl	STL File	CC BY 4.0	http://doi.org/10.17632/776w7sj2f2.1
Roller Module Case.step	CAD file	CC BY 4.0	http://doi.org/10.17632/776w7sj2f2.1
Roller Module Back Plate.stl	STL File	CC BY 4.0	http://doi.org/10.17632/776w7sj2f2.1
Roller Module Back Plate.step	CAD file	CC BY 4.0	http://doi.org/10.17632/776w7sj2f2.1
Roller Module Motor Clip.stl	STL File	CC BY 4.0	https://doi.org/10.17632/776w7sj2f2.1
Roller Module Motor Clip.step	CAD file	CC BY 4.0	https://doi.org/10.17632/776w7sj2f2.1
Roller Module Back Plate (Barrel Jack).stl	STL File	CC BY 4.0	http://doi.org/10.17632/776w7sj2f2.1
Roller Module Back Plate (Barrel Jack).step	CAD file	CC BY 4.0	http://doi.org/10.17632/776w7sj2f2.1
Drive Gear.stl	STL File	CC BY 4.0	http://doi.org/10.17632/776w7sj2f2.1
Drive Gear.step	CAD file	CC BY 4.0	http://doi.org/10.17632/776w7sj2f2.1
Driven Gear.stl	STL file	CC BY 4.0	http://doi.org/10.17632/776w7sj2f2.1
Driven Gear.step	CAD file	CC BY 4.0	http://doi.org/10.17632/776w7sj2f2.1
Roller Module Roller.stl	STL File	CC BY 4.0	http://doi.org/10.17632/776w7sj2f2.1
Roller Module Roller.step	CAD file	CC BY 4.0	http://doi.org/10.17632/776w7sj2f2.1
Module_PCB.kicad_pro	Files	CC BY 4.0	http://doi.org/10.17632/776w7sj2f2.1
Module_PCB_Barrel.kicad_pro	Files	CC BY 4.0	http://doi.org/10.17632/776w7sj2f2.1
Roller_Module_Arduino_Code.ino	Files	CC BY 4.0	http://doi.org/10.17632/776w7sj2f2.1
Final Take Up Module Case.stl	STL File	CC BY 4.0	http://doi.org/10.17632/776w7sj2f2.1
Final Take Up Module Case.step	CAD file	CC BY 4.0	http://doi.org/10.17632/776w7sj2f2.1
Final Take Up Module Back Plate.stl	STL File	CC BY 4.0	http://doi.org/10.17632/776w7sj2f2.1
Final Take Up Module Back Plate.step	CAD file	CC BY 4.0	http://doi.org/10.17632/776w7sj2f2.1
Final Take Up Module Motor Clip.stl	STL File	CC BY 4.0	https://doi.org/10.17632/776w7sj2f2.1
Final Take Up Module Motor Clip.step	CAD file	CC BY 4.0	http://doi.org/10.17632/776w7sj2f2.1
Final Take Up Module Back Plate (Barrel Jack).stl	STL File	CC BY 4.0	http://doi.org/10.17632/776w7sj2f2.1
Final Take Up Module Back Plate (Barrel Jack).step	CAD file	CC BY 4.0	http://doi.org/10.17632/776w7sj2f2.1
Final Take Up Motor Adaptor.stl	STL File	CC BY 4.0	http://doi.org/10.17632/776w7sj2f2.1
Final Take Up Motor Adaptor.step	CAD file	CC BY 4.0	http://doi.org/10.17632/776w7sj2f2.1
Final Take Up Spool Body.stl	STL File	CC BY 4.0	http://doi.org/10.17632/776w7sj2f2.1
Final Take Up Spool Body.step	CAD file	CC BY 4.0	http://doi.org/10.17632/776w7sj2f2.1
Final Take Up Spool Base.stl	STL File	CC BY 4.0	http://doi.org/10.17632/776w7sj2f2.1
Final Take Up Spool Base.step	CAD file	CC BY 4.0	http://doi.org/10.17632/776w7sj2f2.1
Final Take Up Module Stepper Motor Adaptor.stl	STL File	CC BY 4.0	http://doi.org/10.17632/776w7sj2f2.1
Final Take Up Module Stepper Motor Adaptor.step	CAD file	CC BY 4.0	http://doi.org/10.17632/776w7sj2f2.1
Final Take Up Module Stepper Motor Wire Adaptor.stl	STL File	CC BY 4.0	http://doi.org/10.17632/776w7sj2f2.1
Final Take Up Module Stepper Motor Wire Adaptor.step	CAD file	CC BY 4.0	http://doi.org/10.17632/776w7sj2f2.1
Final_Take_Up_Module_Arduino_Code.ino	Files	CC BY 4.0	http://doi.org/10.17632/776w7sj2f2.1
Final_Take_Up_Rast_Module_ESP32.ino	Files	CC BY 4.0	http://doi.org/10.17632/776w7sj2f2.1
Power Junction Case.stl	STL File	CC BY 4.0	http://doi.org/10.17632/776w7sj2f2.1
Power Junction Case.step	CAD file	CC BY 4.0	http://doi.org/10.17632/776w7sj2f2.1
Power Junction Back Plate.stl	STL File	CC BY 4.0	https://doi.org/10.17632/776w7sj2f2.1
Power Junction Back Plate.step	CAD file	CC BY 4.0	http://doi.org/10.17632/776w7sj2f2.1
Power_Junction_PCB.kicad_pro	Files	CC BY 4.0	http://doi.org/10.17632/776w7sj2f2.1
Drying Rig Base.stl	STL File	CC BY 4.0	http://doi.org/10.17632/776w7sj2f2.1
Drying Rig Base.step	CAD file	CC BY 4.0	http://doi.org/10.17632/776w7sj2f2.1
Drying Pulley 606ZZ.stl	STL File	CC BY 4.0	https://doi.org/10.17632/776w7sj2f2.1
Drying Pulley 606ZZ.step	CAD file	CC BY 4.0	http://doi.org/10.17632/776w7sj2f2.1
Drying Pulley Spacer.stl	STL File	CC BY 4.0	http://doi.org/10.17632/776w7sj2f2.1
Drying Pulley Spacer.step	CAD file	CC BY 4.0	https://doi.org/10.17632/776w7sj2f2.1

### 3.1 Baths

• Bath 58 mm: General spin bath with an internal width of 58 mm, designed to stack with the 50 mm bath.
• Bath 50 mm: General spin bath with an internal width of 50 mm, designed to stack with the 58 mm bath.
• Bath 29 mm: General spin bath with an internal width of 29 mm, much smaller fill volume for use with hazardous and/or expensive solvents.
• Coagulation Bath 50 mm Suba_Seal: Specific Coagulation bath with an internal width of 50 mm for use with a number 37 suba seal for horizontal loading of a monofilament spinneret.
• Fibre Clip 58 mm: Clip to hold the forming fibre under the surface of the bath, designed for use with the 58 mm bath.
• Fibre Clip 50 mm: Clip to hold the forming fibre under the surface of the bath, designed for use with the 50 mm bath.
• Fibre Clip 29 mm: Clip to hold the forming fibre under the surface of the bath, designed for use with the 29 mm bath.
• Spinneret Clip 50 mm: Clip for holding a spinneret horizontally at the bottom of the bath, designed for use with the 50 mm bath.

### 3.2 General roller Module

• Roller Module Case: General case for the roller modules.
• Roller Module Back Plate: Back plate for the roller module case, designed to pressure fit into the roller module case.
• Roller Module Motor Clip: Clip designed to fit between the motor and the roller module case to prevent motor rotation during operation.
• Roller Module Back Plate (Barrel Jack): Alternative back plate for use with the barrel jack PCB design.
• Drive Gear: Middle gear for the roller modules, has adaptor for motor shaft.
• Driven Gear: Periphery gears for the roller module.
• Roller Module Roller: Rollers for the roller module, designed to pressure fit onto the shaft of the gears.
• Module PCB: PCB and circuit design for all modules, components and connections labelled, USB-c power input. Module selection is controlled by addition of a solder bridge on the relevant voltage pads (instructions in Section 5.3).
• Module PCB (Barrel Jack): Alternative PCB and circuit design for all modules, components and connections labelled, barrel jack power input. Module selection is controlled by addition of a solder bridge on the relevant voltage pads (instructions in Section 5.3).
• Roller_Module_Arduino_Code.ino: Arduino code for the roller modules.

### 3.3 Take up module

• Final Take Up Module Case: General case for the final take up module.
• Final Take Up Module Back Plate: Back plate for the final take up module case, designed to pressure fit into the final take up module case.
• Final Take Up Module Motor Clip: Clip designed to fit between the motor and the roller module case to prevent motor rotation during operation.
• Final Take Up Module Back Plate (Barrel Jack): Alternative back plate for use with the barrel jack PCB design.
• Final Take Up Motor Adaptor: Adaptor to connect the motor shaft to the spool base, allowing for easy removal and change of spools.
• Final Take Up Spool Body: Body of the take up spool.
• Final Take Up Spool Base: Base of the take up spool, designed to be removable for easy fibre removal.
• Final Take Up Module Stepper Motor Adaptor: Adaptor base plate for a stepper motor rasterization unit.
• Final Take Up Module Stepper Motor Wire Adaptor: Adaptor for inserting wire guide for rasterization.
• Final_Take_Up_Module_Arduino_Code.ino: Arduino code for the final take up module with no optional rasterization unit.
• Final_Take_Up_Rast_Module_ESP32.ino: Arduino code for the final take up module with optional rasterization unit.

### 3.4 Power junction

• Power Junction Case: Case for power junction PCBs.
• Power Junction Back Plate: Back plate for the power junction case, designed for pressure fit.
• Power Junction PCB: PCB and circuit design for the power junction.

### 3.5 Drying Rig

• Drying Rig Base: Base plate for the drying rig.
• Drying Pulley 606ZZ: Pulley for use with the drying rig, designed for use with standard 606ZZ metal bearings and for use with a 6 mm rod.
• Dyring Pulley Spacer: Spacer for the 3D printed pulleys to allow free rotation in either direction.

## 4 Bill of materials summary

Full bill of materials is available on the online repository at https://doi.org/10.17632/776w7sj2f2.1.

## 5 Build instructions

### 5.1 3D printing components

Components Required (from Bill of Materials): PET-G 3D printing filament.

Note: PET-G was chosen due to its chemical compatibility with the solvents and reagents used in the original wet spinning project, it is advised that users research the chemical compatibility of the available 3D printer filaments with their experimental procedure, especially the baths, and to treat all 3D printed components exposed to harsh chemicals as semi-consumable, with routine visual inspection to ascertain need for replacements. Alternatively, glass baths can be used, although this adds to the fabrication cost unless existing baths are available.

#### 5.1.1 3D printer settings

Table 2 gives example 3D printer settings if using PETG.

**Table 2:** 3D printer settings.

Parameter	Value
Material	PETG
Nozzle Size	0.4 mm
Layer Height	0.2 mm
Initial Layer Height	0.2 mm
Line Width	0.4 mm
Initial Layer Line Width	0.4 mm
Wall Thickness	2.4 mm
Wall Line Count	6
Horizontal Expansion	0.0 mm
Top/Bottom Thickness	0.8 mm
Top/Bottom Layers	4
Infill Density	20.0%
Infill Line Distance	6 mm
Infill Pattern	Cubic
Infill Line Multiplier	1
Infill Overlap Percentage	30.0%
Infill Layer Thickness	0.2 mm
Gradual Infill Steps	0
Printing Temperature	235 °C
Printing Temperature Initial Layer	230 °C
Initial Printing Temperature	235 °C
Final Printing Temperature	235 °C
Build Plate Temperature	80 °C
Build Plate Temperature Initial Layer	80 °C
Print Speed	30 mm s^−1^
Infill Speed	30 mm s^−1^
Wall Speed	15 mm s^−1^
Outer Wall Speed	15 mm s^−1^
Inner Wall Speed	15 mm s^−1^
Top/Bottom Speed	15 mm s^−1^
Travel Speed	150 mm s^−1^
Initial Layer Speed	15 mm s^−1^
Retraction Distance	6.5 mm
Retraction Speed	25 mm s^−1^
Combing Mode	Not In Skin
Fan Speed	50%
Initial Fan Speed	0%
Regular Fan Speed at Layer	6
Minimum Layer Time	10.0 s
Minimum Speed	10.0 mm s^−1^

#### 5.1.2 Component printing

**Coagulation Bath.** Print the following files:

• Bath 50 mm.stl
• Fibre Clip 50 mm.stl
• Spinneret Clip 50 mm.stl

Variations: Included in the design file is a bath with a hole in one end designed to be used in combination with a number 37 suba seal, this allows for direct insertion of a mono-filament spinneret through the suba seal during use rather than having to immerse the spinneret and utilise the spinneret clip.

**Hardening/wash bath(s).** Print the following files:

• Bath 58 mm.stl
• Fibre Clip 58 mm.stl x 2.

Print as many hardening/wash baths, of any size, as is required along with 2x fibre clips per bath. Variations: Included in the design files are several different bath sizes along with the associated fibre/spinneret clips.

**Generic Take Up Module.** Print the following files:

• Roller Module Case.stl
• Roller Module Back Plate.stl
• Roller Module Motor Clip.stl
• Drive Gear.stl
• Driven Gear.stl
• Roller Module Roller.stl x2

Variations: Included in the design files are 2 different back plates, one for use with a USB-C adaptor PCB, one with a barrel adaptor PCB.

**Final Take Up Module.** Print the following files:

• Final Take Up Module Case.stl
• Final Take Up Module Back Plate.stl
• Final Take Up Module Motor Clip.stl
• Final Take Up Motor Adaptor.stl
• Final Take Up Spool Body.stl
• Final Take Up Spool Base.stl

Print as many fibre spools and fibre spool bases as desired.

**Optional Rasterization Module.** Print the following file:

• Final Take Up Module Stepper Motor Adaptor.stl
• Final Take Up Module Stepper Motor Wire Adaptor.stl

Variations: Included in the design files are 2 different back plates, one for use with a USB-C adaptor PCB, one with a barrel adaptor PCB.

**Dyring/Drawing Rig.** Print the following file(s):

• Drying Rig Base.stl

Variations: While this paper uses a 3D printed base plate, this does require a 30 x 30 cm build plate for the 3D printer, which is quite large for standard 3D printer setups. If a 30 x 30 cm build plate is not available, a simple block of wood with holes drilled into it at the correct positions will serve just as well. Included in the bill of materials are the pulleys used for the system described, however these are surprisingly expensive, so additionally design files for 3D printed pulleys that utilise standard 606ZZ metal bearings (which are considerably cheaper) has been included. This allows for a less expensive, but more time-consuming route to the same system. If these are to be used, the following files need to be printed for each pulley:

• Drying Pulley 606ZZ.stl
• Drying Pulley Spacer.stl x 2

Once these files have been printed, the 606ZZ bearing can be inserted into the printed pulley, and then when the drying module is assembled, the spacers are added to keep the pulleys apart to allow for free rotation.

**Power junction.** Print the following files:

• Power Junction Case.stl
• Power Junction Back Plate.stl

### 5.2 Arduino coding

Components Required (from Bill of Materials): Arduino Nano Every; Arduino Nano ESP32.

**Fig. 1:**
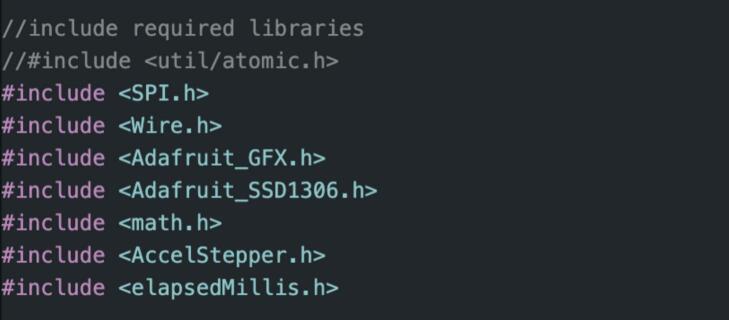
List of arduino libraries to be included.

Firstly, ensure that all required libraries are installed by using the Arduino IDE library manager (Fig. 1), full instructions can be found here: https://docs.arduino.cc/software/ide-v1/tutorials/installing-libraries/.

#### 5.2.1 Generic take up module

Flash Arduino nano every with file Roller_Module_Arduino_Code.ino by connecting the Arduino to a PC via USB, opening the Arduino IDE (either local or web based version), connect to the Arduino, select the correct board and port and click upload.

#### 5.2.2 Final take up module

Flash Arduino nano every with file Final_Take_Up_Module_Arduino_Code.ino by connecting the Arduino to a PC via USB, opening the Arduino IDE (either local or web-based version), connect to the Arduino, select the correct board and port and click upload.

#### 5.2.3 Final take up module with optional rasterization unit

Flash Arduino nano ESP32 with file Final_Take_Up_Rast_Module_ESP32.ino by connecting the Arduino to a PC via USB, opening the Arduino IDE (either local or web-based version), connect to the Arduino, select the correct board and port and click upload.

#### 5.2.4 Arduino code editing

**Fig. 2:**
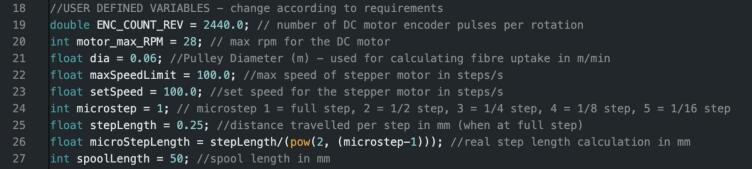
User defined variables for the Arduino code.

With adequate knowledge, the user can change anything written in the design files, depending on their need. Comments have been added to assist with understanding the code and re-flashing the Arduino with the original file is always a backup in-case something goes wrong. There are a few variables that a user might wish to change in the Arduino code without completely altering the code. These variables have been grouped near the start of the Arduino code file and are clearly labelled as ‘USER DEFINED VARIABLES’ (Fig. 2). A brief explanation of each variable is given below:

All Modules:

**ENC_COUNT_REV**: The number of encoder pulses per motor revolution. This should only be changed if a motor with a different gearbox is used or if different magnetic encoder disks are used. The current value is for 10-pole magnetic disks and a 488:1 gearbox. If a different disk or a motor with a different gearbox is used the number of pulses per revolution is given by the equation:

$$\mathrm{ENCODERPULSESPERREVOLUTION}=\frac{\mathrm{numberofencoderpoles}}{2}\times gearratio$$

**Motor_max_RPM**: the maximum RPM for the installed motor. Should only be changed if a motor with a different gearbox is used.

**Dia:** diameter of the pulley used (in the case of the roller module) or the diameter of the spool (in the case of the final take up module). Should only be changed if different pulleys/spools are used.

Final Take Up Module with Rasterization Only:

**maxSpeedLimit**: maximum speed for the stepper motor in steps. Users may want to adjust this based on their specific use case.

**Microstep**: sets the microstep for the stepper motor, 1 = full step, 2 = half step, 3 = quarter step, 4 = 1/8 step, 5 = 1/16 step. The user can change this value to slow down or speed up the effective rasterization of the fibre via the stepper motor.

**stepLength**: the step length of the stepper motor when at full step. Should only be changed if using a different stepper motor.

**spoolLength**: The length of the spool used for the final take up module. This value directly controls the rasterization length and so can be adjusted to rasterize over a shorter or longer distance. If using a shorter or longer spool this should be updated accordingly.

### 5.3 PCBs

Components Required (from Bill of Materials):

• Module PCB
• Power Junction PCB
• USB-C Breakout
• Arduino Nano Every
• Arduino Nano ESP32
• A4988 Stepper Motor Driver
• MP6550 DC Motor Driver
• Barrel Jack
• Header Pins
• Jumper Wires

Firstly, either directly print the PCBs or have them printed by a third party (such as JLCPCB).

#### 5.3.1 Take up modules

**Fig. 3:**
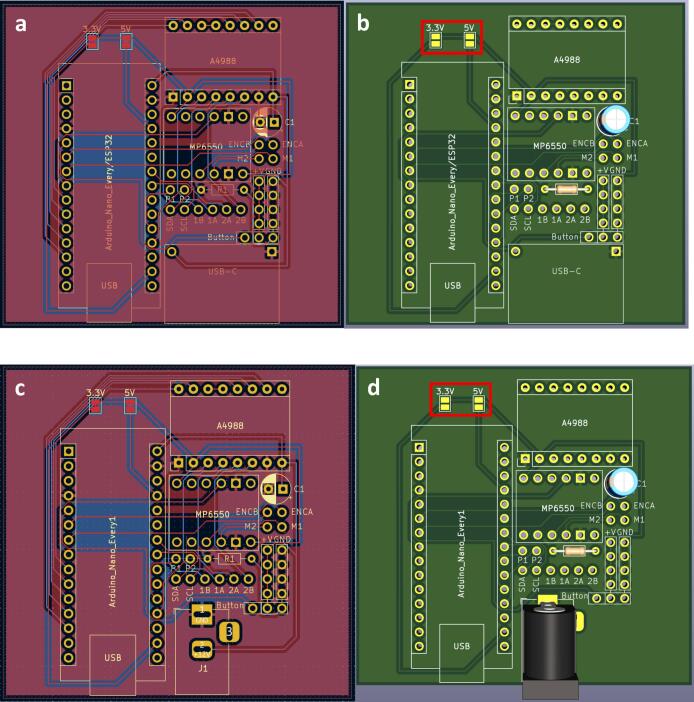
PCB design (a) and visual mock up (b) for USB-C version. PCB design (c) and visual mock up (d) for barrel jack powered versions.

**Fig. 4:**
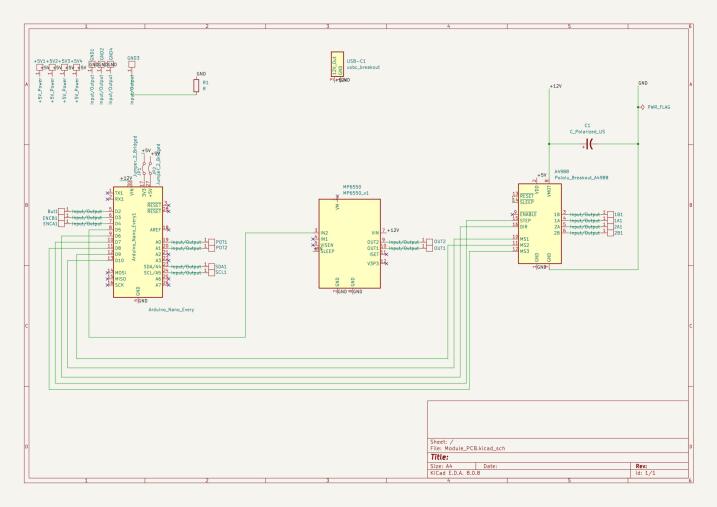
Circuit diagram for module PCBs.

To streamline the design, only one PCB design is required regardless of which module is being made (which lowers costs when printing PCBs). So firstly, determine which module the PCB is going to be used for. For the generic take up module, or final take up module without the optional rasterization, add a solder bridge at the 5 V label (this is due to the Arduino nano Every logic circuit running at 5 V). For the final take up module with rasterization add a solder bridge at the 3.3 V label (this is due to the Arduino nano ESP32 logic circuit running at 3.3 V). The default PCB is powered by a USB-C input (Fig. 4) which works in conjunction with the power junction box. However, a PCB version which replaces the USB-C breakout board with a standard barrel jack has also been included, which allows for individual modules to be powered directly by a 12 V plug adaptor as listed in the bill of materials (Fig. 3). The only other difference to take into account is the need to print the correct back plate for the module (as labelled in the production files).

#### 5.3.2 Generic take up module

**Fig. 5:**
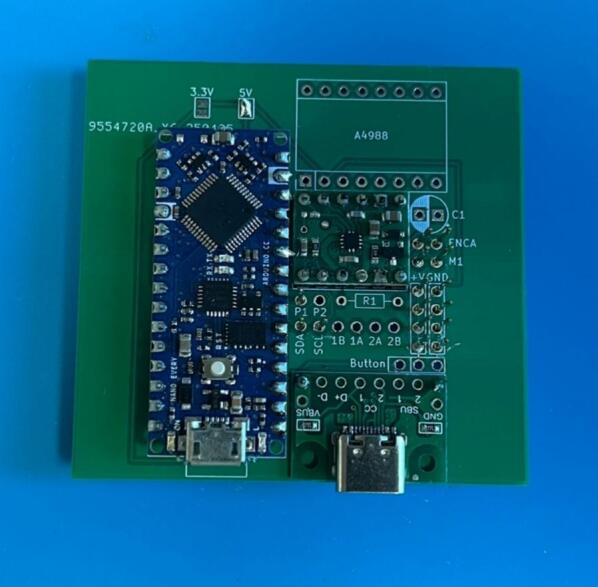
PCB set up for the take up module with peripheries and header pins added.

Ensure a solder bridge is in place at the point labelled 5 V on the PCB. Then solder the Arduino nano every, the USB-C breakout board and the MP6550 DC motor driver onto the board (aligning the GND pins on the board (denoted by a square solder pad) with the GND pins on the components) (Fig. 5).

#### 5.3.3 Final take up module with rasterization

**Fig. 6:**
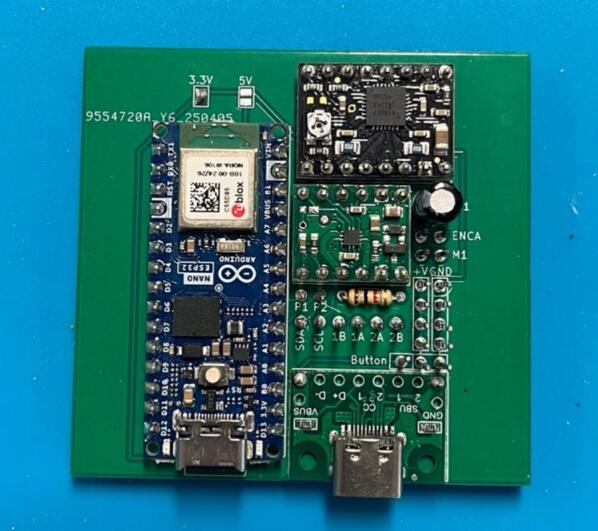
PCB set up for the final take up module with optional rasterization unit, with peripheries and header pins added.

Ensure a solder bridge is in place at the point labelled 3.3 V on the PCB. Then solder the Arduino nano ESP32, the USB-C breakout board, the MP6550 DC motor driver and the A4988 stepper motor driver, (aligning the GND pins on the board (denoted by a square solder pad) with the GND pins on the components) (Fig. 6).

#### 5.3.4 Power junction box

**Fig. 7:**
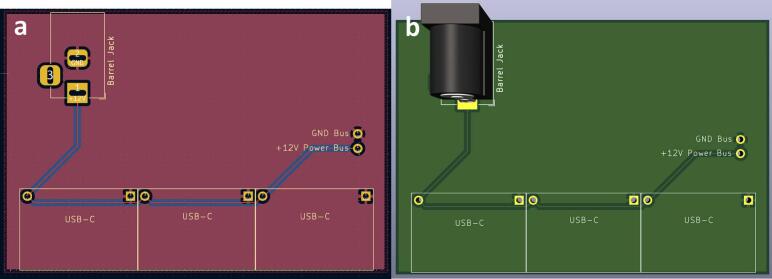
PCB design (a) and visual mock up (b) for the power junction circuit.

**Fig. 8:**
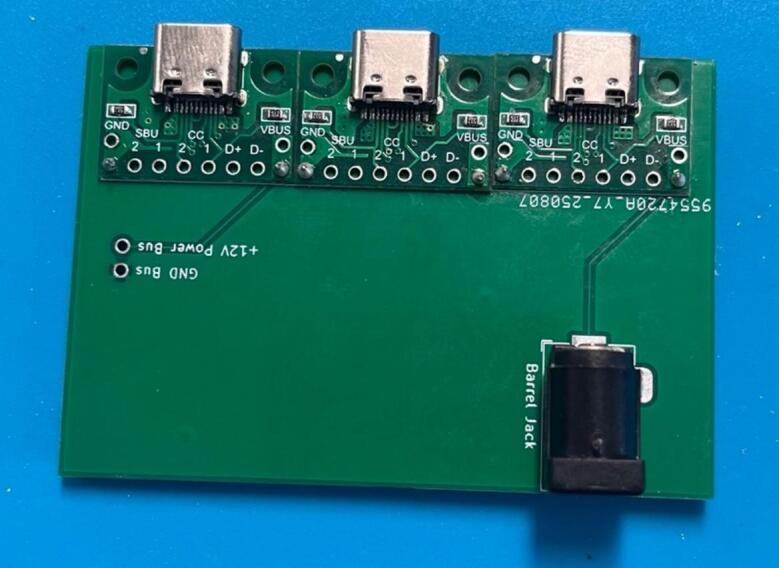
PCB for the power junction with peripheries added.

Solder the USB-C breakout boards and the barrel jack onto the boards (Fig. 7), aligning the GND on the board (denoted by a square solder pad) with the GND on the components (Fig. 8). If using two boards for the full capacity of the power junction box, solder a short jumper cable between the two boards connecting the GND bus of one to the GND bus of the other, and the 12 V bus of one to the 12 V bus of the other.

### 5.4 Component preparation and Assembly

Components Required (from Bill of Materials):

• Motor
• Motor Encoder
• LCD Screen
• 10 turn potentiometer
• 1 turn potentiometer
• Button
• 1 kOhm resistor
• 100 uF capacitor
• Jumper wires
• USB-C to USB-C cable
• Pulleys (unless self-fabricating)

#### 5.4.1 Generic take up module

**Fig. 9:**
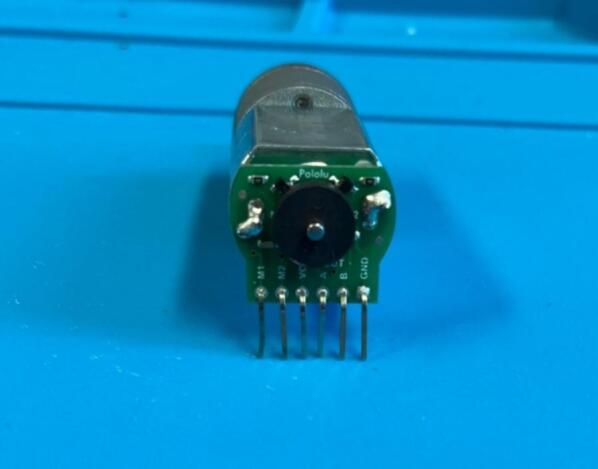
Motor encoder and header pin assembly.

Motor: Solder the encoder breakout board onto the motor and push the magnetic disk onto the rear motor shaft. Either solder header pins onto the encoder breakout board, and ensure they are at right angles to the motor (Fig. 9), or solder wires directly onto the board. Ensure wires are colour coded or labelled for ease of wiring later.

Potentiometer: Either solder header pins onto the potentiometer pins, or solder wires directly onto the board. Ensure wires are colour coded or labelled for ease of wiring later.

**Fig. 10:**
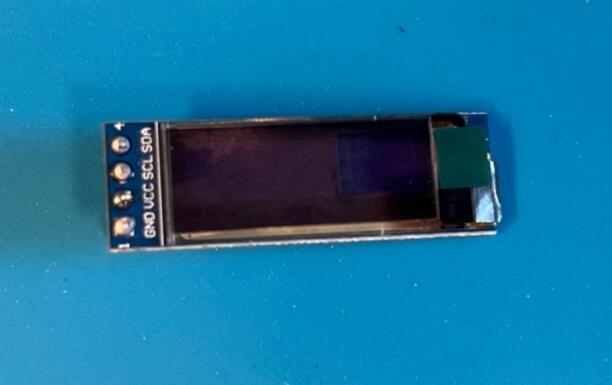
Screen with header pins.

Screen: Either solder header pins onto the board (Fig. 10), or solder wires directly onto the board. Ensure wires are colour coded or labelled for ease of wiring later.

**Fig. 11:**
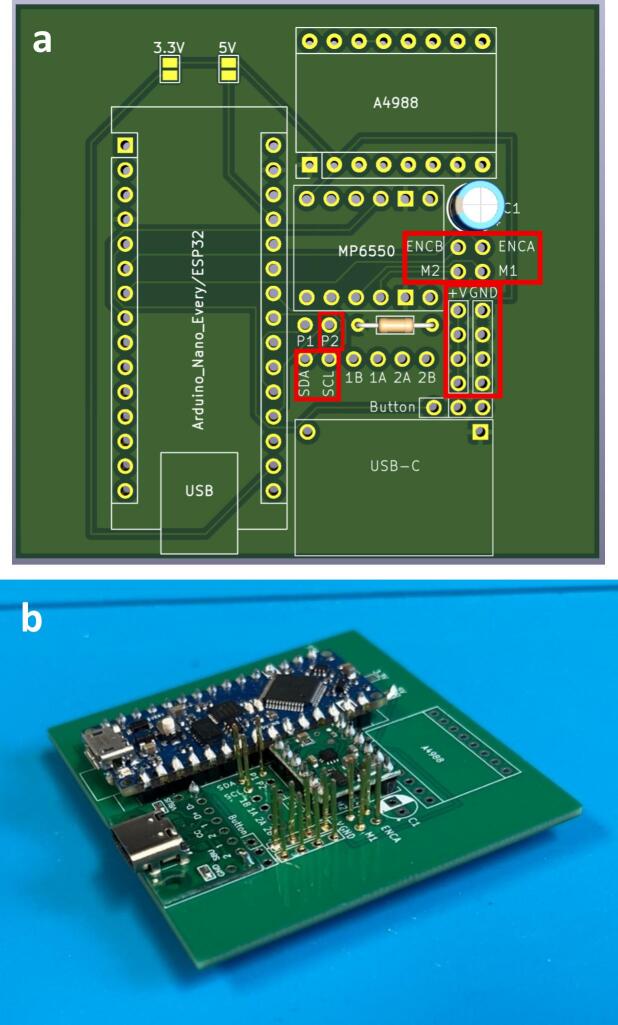
Jumper pads needed for roller module (highlighted in red) (a) and full board with header pins added (b). (For interpretation of the references to colour in this figure legend, the reader is referred to the web version of this article.)

Connections: Either solder header pins onto the following jumper pads labelled: p2, SDA, SCL, ENCB, ENCA, M2, M1, +V x 4 (not including Button + V) and GND x 4 (not including Button GND) (Fig. 11) or solder component wires directly into the board at the relevant jumper pads. Ensure wires are colour coded or labelled for ease of wiring later.

**Fig. 12:**
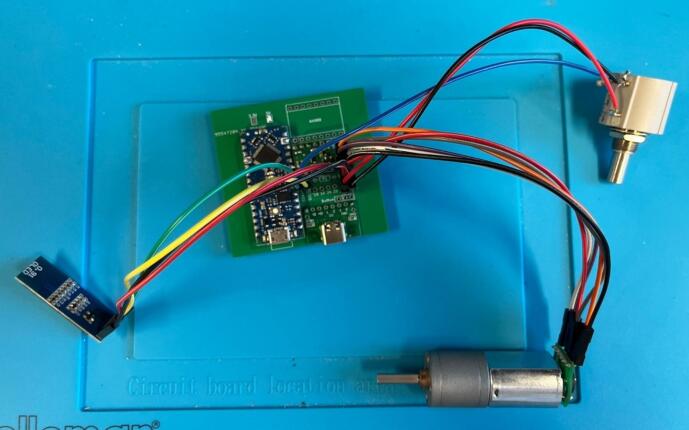
Fully assembled circuit ready to be installed in the case.

Wiring: The below example has been wired with all female-female jumper cables, with the exception of the potentiometer cables where one end of the cable has been stripped and soldered directly onto the potentiometer connections (Fig. 12). Wires have been colour coded in the example in Table 3:

**Table 3:** Wiring colours for different connections.

Connection	Wire Colour
+5V Logic	Red
Ground	Black
Potentiometer Wiper Pin	Blue
Display SCL Connection	Yellow
Display SDA Connection	Green
Motor Encoder A	Orange
Motor Encoder B	Grey
Motor Input 1	White
Motor Input 2	Purple

It is not a requirement to have the cables coloured this way, so long as there is differentiation between the different connections.

Table 4 shows what to connect (via either female jumper cable, or solder):

**Table 4:** Details for wiring to connections.

Connection	Colour	PCB Pad
Potentiometer 1 + 5 V in	Red	+V (any)
Potentiometer 1 GND	Black	GND (any)
Potentiometer 1 Wiper	Blue	P2
Display + 5 V in	Red	+V (any)
Display GND	Black	GND (any)
Display SCL Connection	Yellow	SCL
Display SDA Connection	Green	SDA
Motor + 5 V in (VCC)	Red	+V (any)
Motor GND	Black	GND (any)
Motor Encoder A	Orange	ENCA
Motor Encoder B	Grey	ENCB
M1	White	M1
M2	Purple	M2

**Fig. 13:**
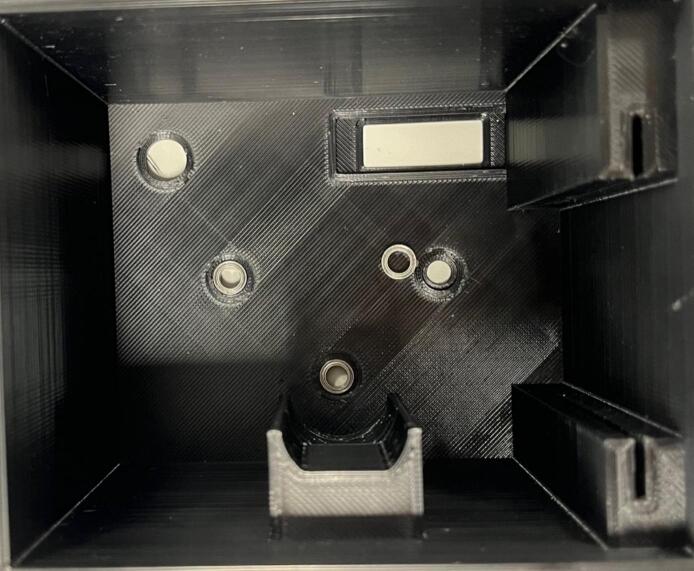
Roller bearings being inserted into their designated grooves.
• Insert the drive cog through the central hole and the driven cogs into the two periphery holes and affix the rollers onto the shafts from the outside of the case (using a drop of hot glue or other adhesive if needed) (Fig. 14).

Assembly:

• Using a drop of hot glue (or other adhesive) affix the screen onto the view port on the inside of the roller module case.
• Insert the potentiometer through the designated hole and affix by screwing the fastener but onto the outside of the case.
• Insert three roller bearings into the designated grooves on the inside of the case (Fig. 13).

**Fig. 14:**
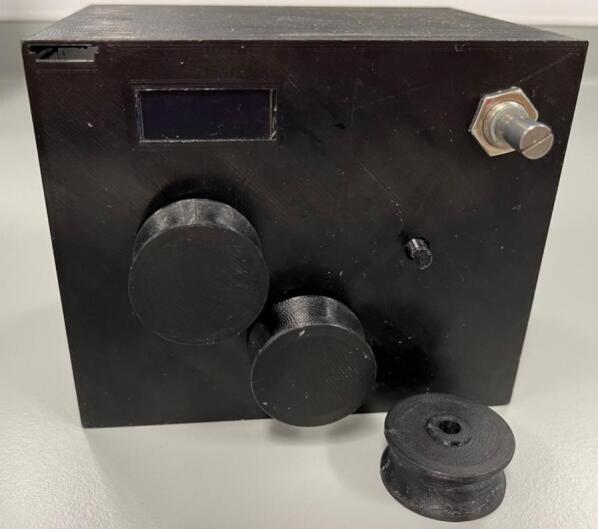
Rollers being attached to the cog shafts.
• Insert the motor into the motor holder and ensure the drive shaft is firmly placed into the designated slot on the drive cog.
• Place the motor clip into the case ensuring a tight fit to prevent motor movement during operation.
• Insert the PCB into the designated slot and ensure it is securely placed (Fig. 15).

**Fig. 15:**
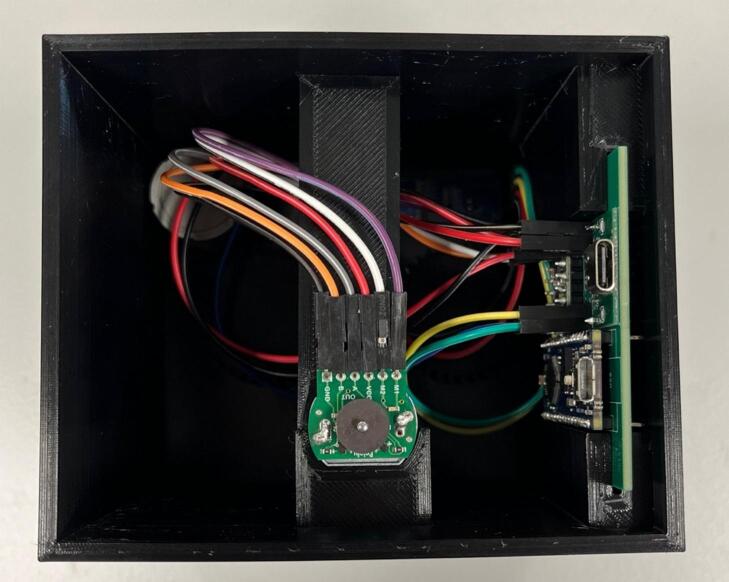
Fully assembled roller module with motor clip installed.
• Ensure all wired connections are still in place.
• Push the back plate into position ensuring USB-C port is accessible, can use hot glue or blu-tac to secure back plate if needed.

#### 5.4.2 Final take up module

Motor: Solder the encoder breakout board onto the motor and push the magnetic disk onto the rear motor shaft. Either solder header pins onto the encoder breakout board, or solder wires directly onto the board. Ensure wires are colour coded or labelled for ease of wiring later.

Potentiometer: Either solder header pins onto the potentiometer pins, or solder wires directly onto the board. Ensure wires are colour coded or labelled for ease of wiring later.

Screen: Either solder header pins onto the board, or solder wires directly onto the board. Ensure wires are colour coded or labelled for ease of wiring later.

**Fig. 16:**
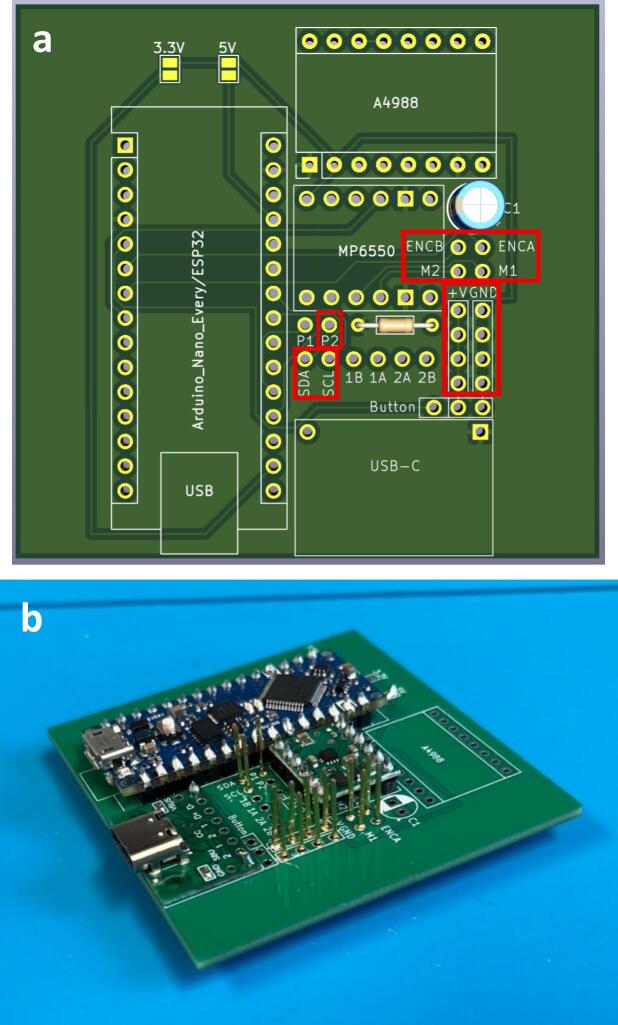
Jumper pads needed for final take up module (highlighted in red) (a) and full board with header pins added (b). (For interpretation of the references to colour in this figure legend, the reader is referred to the web version of this article.)

Connections: Either solder header pins onto the following jumper pads labelled: p2, SDA, SCL, ENCB, ENCA, M2, M1, +V x 4 (not including Button + V) and GND x 4 (not including Button GND) (Fig. 16) or solder component wires directly into the board at the relevant jumper pads. Ensure wires are colour coded, or labelled for ease of wiring later.

Wiring:

An example of wire colour coding is given in Table 5.

**Table 5:** Wiring colours for different connections.

Connection	Wire Colour
+5V Logic	Red
Ground	Black
Potentiometer Wiper Pin	Blue
Display SCL Connection	Yellow
Display SDA Connection	Green
Motor Encoder A	Orange
Motor Encoder B	Grey
Motor Input 1	White
Motor Input 2	Purple

It is not a requirement to have the cables coloured this way, so long as there is differentiation between the different connections.

Table 6 shows what to connect (via either female jumper cable, or solder):

**Table 6:** Details for wiring to connections.

Connection	Colour	PCB Pad
Potentiometer 1 + 5 V in	Red	+V (any)
Potentiometer 1 GND	Black	GND (any)
Potentiometer 1 Wiper	Blue	P2
Display + 5 V in	Red	+V (any)
Display GND	Black	GND (any)
Display SCL Connection	Yellow	SCL
Display SDA Connection	Green	SDA
Motor + 5 V in (VCC)	Red	+V (any)
Motor GND	Black	GND (any)
Motor Encoder A	Orange	ENCA
Motor Encoder B	Grey	ENCB
M1	White	M1
M2	Purple	M2

**Fig. 17:**
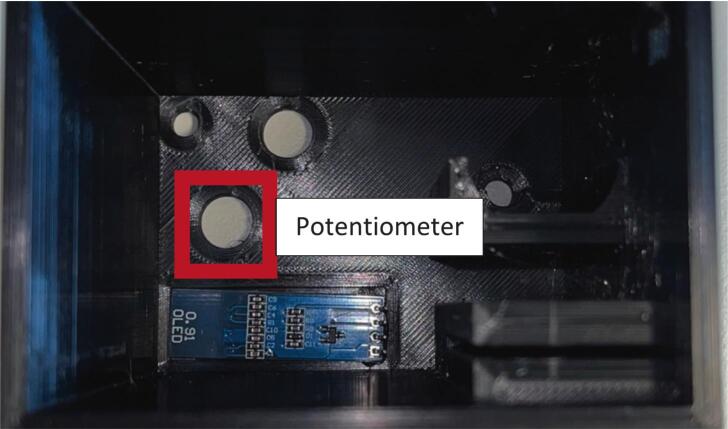
Screen glued into the case and potentiometer hole designation.
• Insert the motor into the motor holder and ensure the drive shaft is accessible from the front face of the case.
• Insert the motor drive shaft into the motor adaptor and ensure a tight and secure fit (can use a drop of hot glue if needed).
• Place the motor clip into the case ensuring a tight fit to prevent motor movement during operation.
• Insert the PCB into the designated slot and ensure it is securely placed.
• Double check all wired connections are still in place.
• Push the back plate into position ensuring USB-C port is accessible, can use hot glue or blu-tac to secure back plate if needed.

Assembly:

• Using a drop of hot glue (or other adhesive) affix the screen onto the view port on the inside of the roller module case.
• Insert the potentiometer through the designated hole and affix by screwing the fastener nut onto the outside of the case (Fig. 17).

#### 5.4.3 Final take up module with optional rasterization unit

Motor: Solder the encoder breakout board onto the motor and push the magnetic disk onto the rear motor shaft as previously shown (Fig. 9).

Either solder header pins onto the encoder breakout board, or solder wires directly onto the board. Ensure wires are colour coded or labelled for ease of wiring later.

Potentiometer 1: Either solder header pins onto the potentiometer pins, or solder wires directly onto the board. Ensure wires are colour coded or labelled for ease of wiring later.

Potentiometer 2: Either solder header pins onto the potentiometer pins, or solder wires directly onto the board. Ensure wires are colour coded or labelled for ease of wiring later.

Button: Either solder header pins onto the button pins, or solder wires directly onto the board. Ensure wires are colour coded or labelled for ease of wiring later.

Screen: Either solder header pins onto the board, or solder wires directly onto the board. Ensure wires are colour coded or labelled for ease of wiring later.

Resistor: Solder a 1 kΩ resistor where indicated with ‘R1′ on the PCB.

Capacitor: Solder a 100uF capacitor onto the PCB where indicated, ensuring that the correct orientation of anode and cathode (as denoted by the + printed on the PCB).

Stepper Motor Driver: Perform current limiting on the A4988 stepper motor driver, this can be done by following the instructions provided by the motor driver supplier, e.g. https://www.pololu.com/product/1182.

**Fig. 18:**
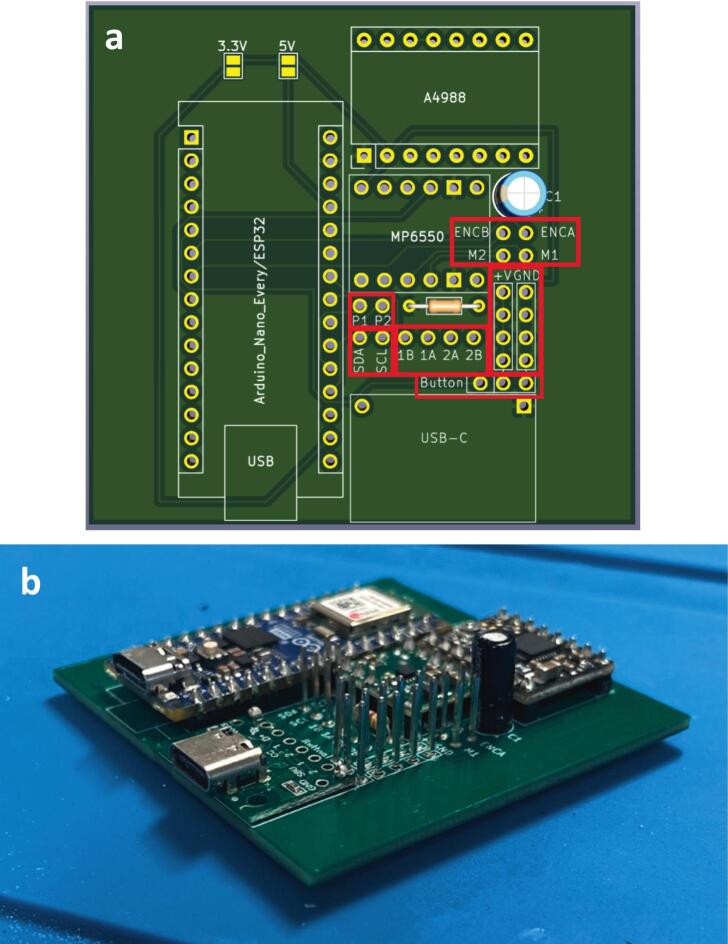
Jumper pads needed for final take up module with optional rasterization module (highlighted in red) (a) and full board with header pins added (b). (For interpretation of the references to colour in this figure legend, the reader is referred to the web version of this article.)

Connections: Either solder header pins onto all remaining jumper pads (labelled: p1, p2, SDA, SCL, 1B, 1A, 2A, 2B, ENCB, ENCA, M2, M1, +V x 5, GND x 5 and Button) (Fig. 18) or solder component wires directly into the board at the relevant jumper pads. Ensure wires are colour coded, or labelled for ease of wiring later.

**Fig. 19:**
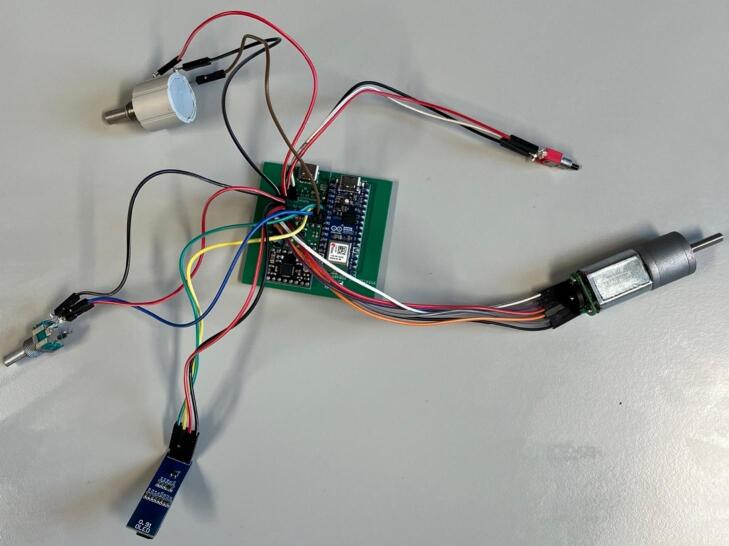
Fully assembled circuit for the final take up with rasterization ready for installation into the case.

Wiring: The below example has been wired with all female-female jumper cables (Fig. 19). Wires have been colour coded in the below in Table 7.

**Table 7:** Wiring colours for different connections.

Connection	Wire Colour
+3.3 V Logic	Red
Ground	Black
Potentiometer 1 Wiper Pin	Blue
Potentiometer 2 Wiper Pin	Brown
Button	White
Display SCL Connection	Yellow
Display SDA Connection	Green
Motor Encoder A	Orange
Motor Encoder B	Grey
Motor Input 1	White
Motor Input 2	Purple

It is not a requirement to have the cables coloured this way, so long as there is differentiation between the different connections.

Table 8 shows what to connect (via either female jumper cable, or solder):

**Table 8:** Details for wiring to connections.

Connection	Colour	PCB Pad
Potentiometer 1 + 3.3 V in	Red	+V (any except button)
Potentiometer 1 GND	Black	GND (any except button)
Potentiometer 1 Wiper	Blue	P2
Potentiometer 2 + 3.3 V in	Red	+V (any except button)
Potentiometer 2 GND	Black	GND (any except button)
Potentiometer 2 Wiper	Brown	P1
Button 3.3 V in	Red	+V Button
Button GND	Black	GND Button
Button Info	White	Button
Display + 3.3 V in	Red	+V (any except button)
Display GND	Black	GND (any except button)
Display SCL Connection	Yellow	SCL
Display SDA Connection	Green	SDA
Motor 3.3 V in (VCC)	Red	+V (any except button)
Motor GND	Black	GND (any except button)
Motor Encoder A	Orange	ENCA
Motor Encoder B	Grey	ENCB
M1	White	M1
M2	Purple	M2

**Fig. 20:**
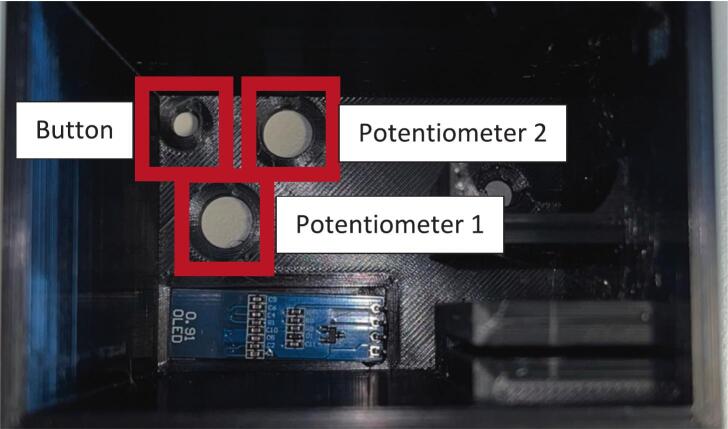
Screen glued into case and potentiometer 1, 2 and button hole designation.
• Using a drop of hot glue (or other adhesive) affix the Final Take Up Module Stepper Wire Adaptor to the stepper motor (as shown in Fig. 21).

Assembly:

• Using a drop of hot glue (or other adhesive) affix the screen onto the view port on the inside of the roller module case (Fig. 20).

**Fig. 21:**
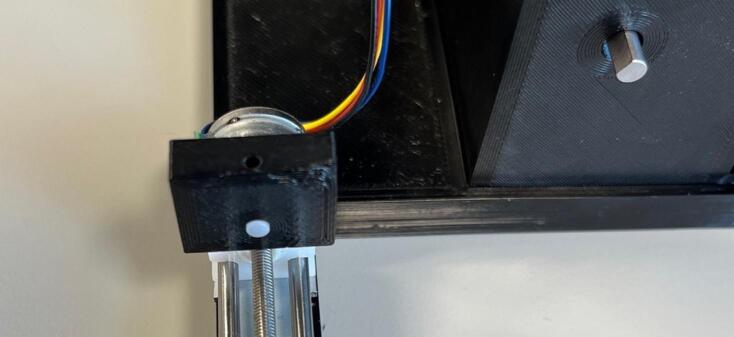
Stepper motor wire adaptor glued onto the stepper motor.
• Insert potentiometer 1 through the designated hole and affix by screwing the fastener nut onto the outside of the case (Fig. 20).
• Insert potentiometer 2 through the designated hole and affix by screwing the fastener nut onto the outside of the case (Fig. 20).
• Insert the button through the designated hole and affix by screwing the fastener nut onto the outside of the case (Fig. 20).
• Insert the motor into the motor holder and ensure the drive shaft is accessible from the front face of the case.
• Insert the motor drive shaft into the motor adaptor and ensure a tight and secure fit (can use a drop of hot glue if needed).
• Place the motor clip into the case ensuring a tight fit to prevent motor movement during operation (Fig. 22).

**Fig. 22:**
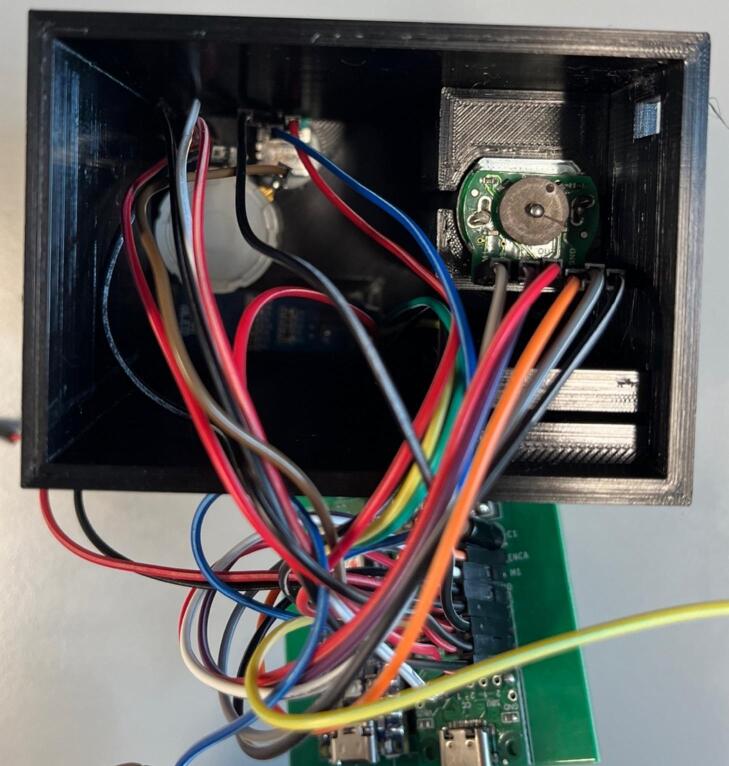
Both potentiometers, button, screen and motor installed into the case.
• Insert the stepper motor wires through the designated hole in the side of the module and connect to the designated pins on the PCB either via inserting header pins into the stepper motor connector, or by cutting the original connector off and soldering header pins directly to the wires, then connect to the PCB via either a female to female jumper wire, or via direct solder.
• Place the stepper motor on the adaptor so that the pins slot into the corresponding holes (Fig. 23).

**Fig. 23:**
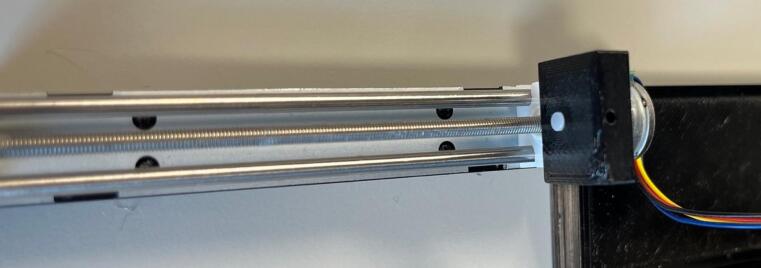
Stepper motor installed onto the adaptor.
• The wire guide for the rasterization unit can be made with any soft wire, or even a paperclip that has been bent into a ‘U’ shape.
• Insert the PCB into the designated slot and ensure it is securely placed.
• Double check all wired connections are still in place (Fig. 24).

**Fig. 24:**
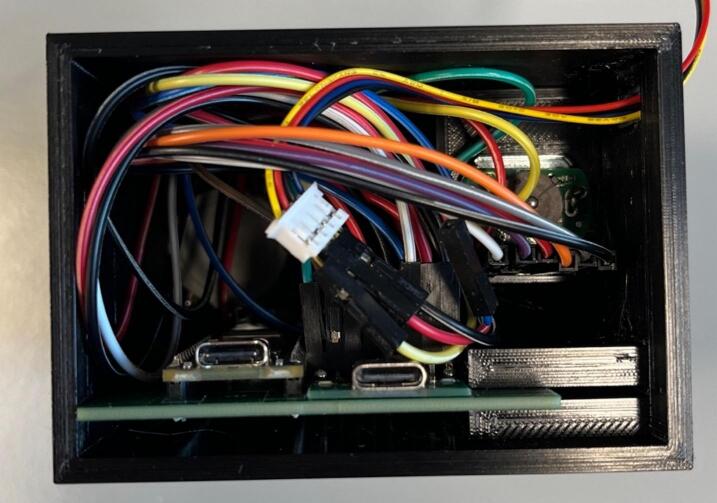
PCB installed into the case, and stepper motor wires threaded through and connected.
• Push the back plate into position ensuring USB-C port is accessible, can use hot glue or blu-tac to secure back plate if needed (Fig. 25).

**Fig. 25:**
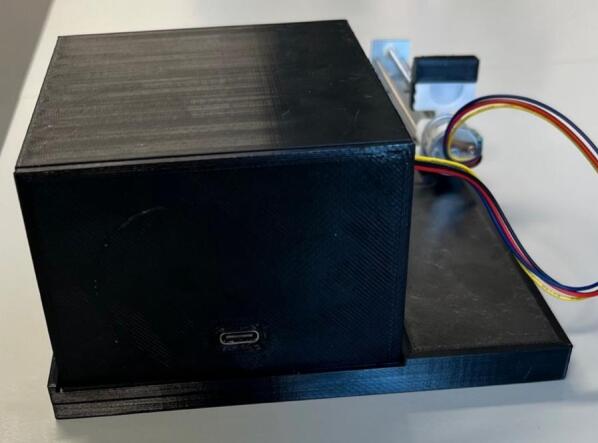
Back plate installed.

#### 5.4.4 Drying/drawing rig

**Fig. 26:**
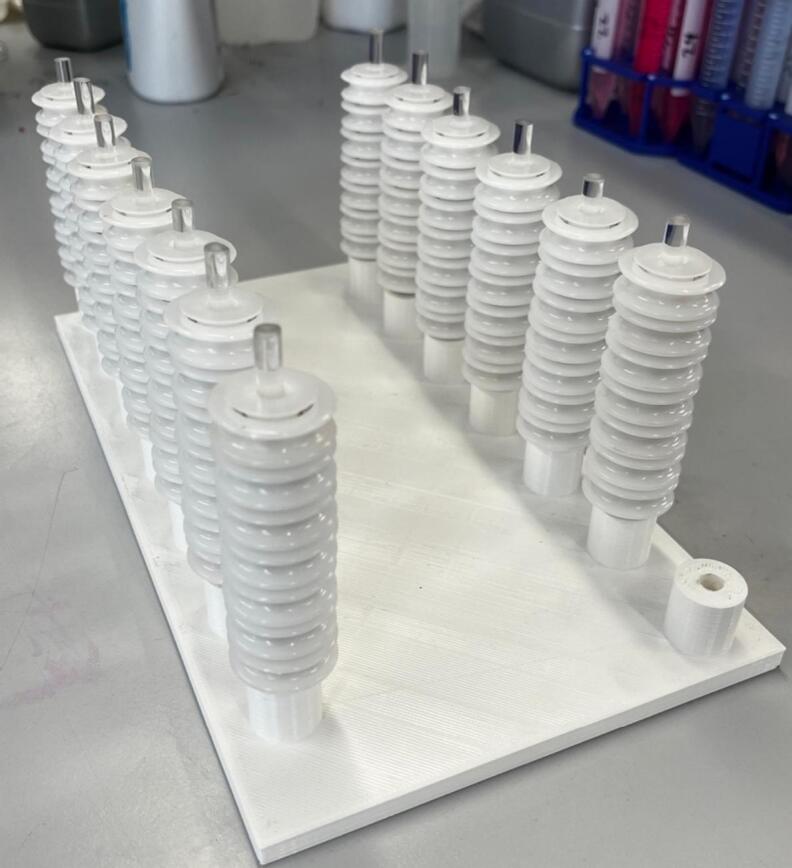
Fully assembled drying module.

Once the base plate has been 3D printed or fabricated, cut the 6 mm acrylic rods down to the size required (calculated by the number of ‘passes’ required for drying times the height of the pulleys used). Insert the rods into the designated holes (using a drop of hot glue if needed to ensure a secure fit). Place the requisite number of pulleys (either purchased or printed) onto each rod (Fig. 26).

## 6 Operation instructions

The wet spinning process will be different for each polymer used, but below is a generalised method for setting up and running the system using casein as an example fibre following the procedure used for a previously published novel *in-situ* colouration method using the system [11].

### 6.1 Non-hardware methods

#### 6.1.1 Dope preparation

Hydrate 45 g (22.5 wt%) casein powder in 143 g distilled water by stirring at 500 rpm for 2 h in a sealed container. Add 12 g of 25 wt% NaOH solution to the container and continue stirring at 140 rpm for 4 h. Leave container sealed for 16 h to age the dope.

#### 6.1.2 Coagulation bath preparation

Dissolve 120 g of MgSO_4_ and 120 g of Na_2_SO_4_ in 600 g distilled water. Slowly add 100 g of concentrated H_2_SO_4_ to the solution under constant stirring. Store in a sealed container until required.

#### 6.1.3 Dyebath preparation

Slowly add 25 g of concentrated H_2_SO_4_ to 475 g of distilled water (this can be stored ready for use). Add 0.5 g of blackcurrant extract [12] to the solution. Stir at ∼500 rpm until dye has completely solubilised. Use dyebath that day.

#### 6.1.4 Experimental set up

**Fig. 27:**
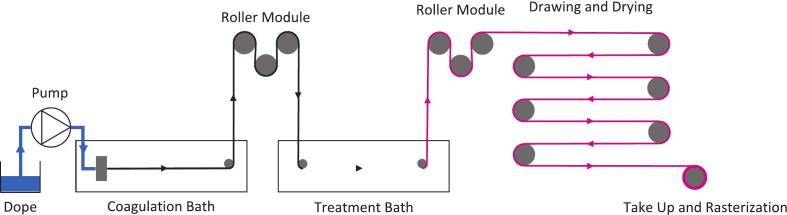
Schematic diagram of the spinning process (in this case inclusive of an in-situ dyeing step).

**Fig. 28:**
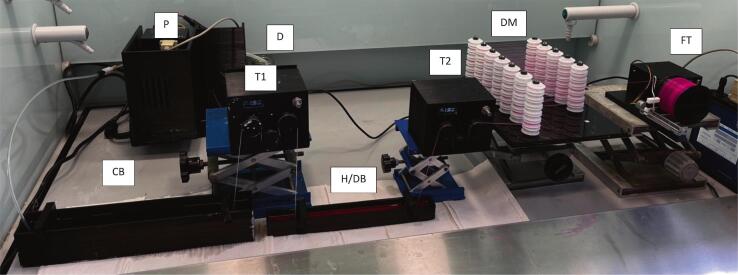
Key: D: spin dope; P: pump; CB: coagulation bath; T1/2: take up modules; H/DB: harden/dyebath; DM: drying module; FT: final take up module.

6.1.5 Rig Set Up (as per Fig. 27 and 28).

**Fig. 29:**
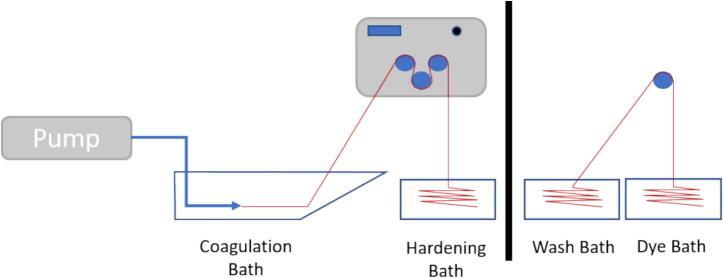
Schematic for the simplest setup for the spinning set up.

**Fig. 30:**
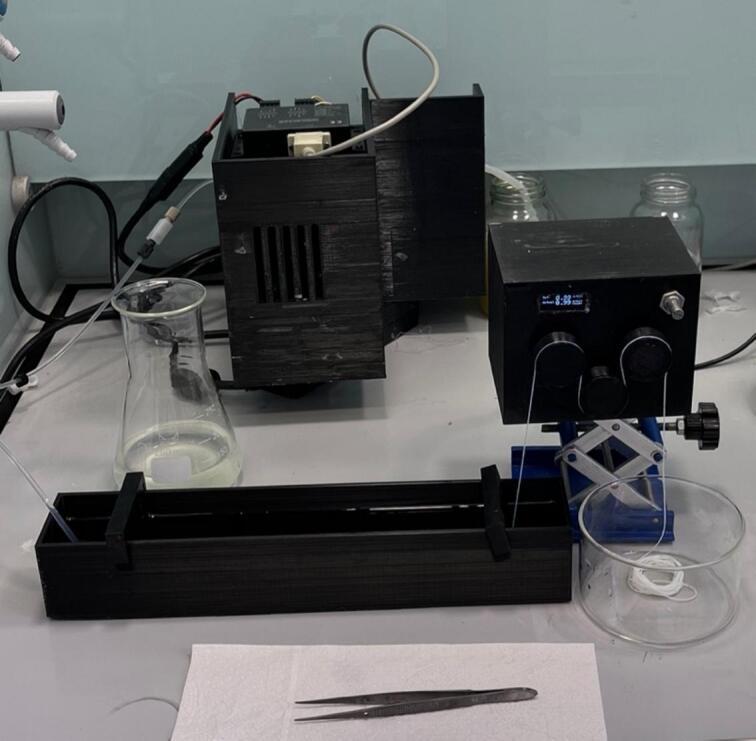
Experimental set up showing the simplest set up for spinning.

• Set up spin pump and connect spinneret to pump via appropriate tubing.
• Set up CB to the far left of the fume hood.
• Set up T1 on top of a lab jack so that T1 is overhanging the end of CB and the beginning of H/DB
• Set up H/DB directly under T1
• Set up T2 on a lab jack so that it is overhanging the end of H/DB
• Set up DM on a lab jack so that the top of the final roller of T2 is level with the second pulley from the bottom on the DM
• Set up FT on a lab jack so that the top of the bobbin is level with the top pulley of DM
• Set up the USB-c junction box by plugging in the provided plug and connecting the barrel adaptor to the back of the junction box, connect up T1, T2 and FT to the junction box using the provided USB-C to USB-C cables
• Ensure all electronics are working by turning on the power and testing each one individually
• Alternatively, the rig can be set up as shown in Fig. 29 with only a single take up module with the fibre allowed to convolute into a bath, or onto a collection dish, before being manually wound into secondary baths/onto a glass cylinder to dry Fig. 30.

#### 6.1.5 Spinning Procedure

Pump is primed by flushing with ∼500 mL distilled water through it at 20 mL min^−1^. 100 mL of dilute NaOH (dope dissolution solvent) is then flushed through the pump system to prevent the protein crashing out of solution in the pipes/pump head. Dope is run through the pump at a rate of 1 mL min^−1^ until observed extruding from the spinneret. Flow rate is then lowered to 0.5 mL min^−1^ and dope is flushed through the system until no air bubbles are observed in the outlet tubing. Spinneret immersed into coagulation bath and held at the bottom of the bath *via* the spinneret clip. Extruded fibre is carefully guided through the coagulation bath and onto the take up rollers (running at 1.25 m min^−1^) using a pair of tweezers, a fibre clip is used to ensure the fibre is kept under the surface of the bath for the entire length. The fibre is then guided through the second bath (the constituents of depend on the experiment), held under the surface for the entire length of the bath with the clips, and onto the third take up roller held at 1.5 m min^−1^. The first ∼30 m of fibre spun is discarded and allowed to collect onto the fume hood floor or into a collection vessel (this is to ensure concentration of dope in the pump has stabilised and to allow any remaining air bubbles/inconsistencies in the pump line to be removed). The fibre is then taken through the drying module on a convoluted path backwards and forwards to allow the fibre to air dry before being wound onto the final take up roller, which is held at between 3.0 m min^−1^ and 4.5 m min^−1^ depending on the experiment, allowing a large draw ratio on the drying fibre.

The method used by the authors for taking the fibre through the drying module is as follows: wind the fibre onto the end of the glass rod at least 5-10x to prevent slipping; while maintaining constant tension, guide the fibre onto the second pulley from the bottom of the DM; wind the fibre around this pulley, then back along a straight path onto the opposite pulley, and wind around this one; repeat all the way to the back of the DM. Once at the final pulley, once wound around it, take the fibre onto the pulley third from the bottom (fibre path will be at a slight angle) and continue back to the front of the module on the 3rd from bottom pulleys. Continue backwards and forwards until fibre is on the top pulleys (6x backwards and forwards) or until fibre is dry (depends on the fibre properties and can be as little as once backwards and forwards). Once on the final pulley at the front of the DM, take the fibre and wrap carefully around the final take up module, keeping hold of the fibre until several rotations have been completed and the fibre does not slip. Trim excess fibre and leave to wind for a few revolutions before starting rasterization. Note: Tension is of key importance during this process, a balance has to be struck maintaining enough tension so that the fibre doesn’t ‘sag’ at the inlet but not too much tension that the fibre snaps (which results in having to start from the beginning). Practice is key to learn the exact tension required. When taking the fibre through the rig, rotating the glass rod occasionally to wind more of the fibre onto the rod removes the localised stress point where the fibre joins onto the rod (which is a common place for the fibre to snap). To start rasterization, manually move the module so that the fibre is winding directly above the stepper motor, then insert the wire guide so that the wire is either side of the fibre being wound. Set rasterization going by clicking the button on the module, adjusting the rasterization speed via the second potentiometer. Rasterization is only required for long spinning runs, due to the fact that if the fibre is left to wind over the same place on the spool the perceived diameter the fibre is being wound round increases. As the set speed is the speed at the surface of the spool this means that the speed of uptake slowly increases, which in turn increases tension leading to non-uniform draw ratios and eventually to fibre breakage. By rasterizing the winding, it greatly slows down the increase in diameter, allowing for a much longer spinning time before the change in uptake speed has an effect.

The fibre can be kept on the original spools, or wound onto carboard bobbins or cones depending on the need.

Electrical Safety Note:

Before using this system, a full COSHH and risk assessment should be performed as per the parent organisations guidelines. Below are some general suggestions for safely using the system, but should not be used as a replacement for generation of relevant COSHH and risk assessments:

• All electrical components should be kept within the polymer cases, with the backs securely fastened to reduce risk, these enclosures help prevent accidental contact with live parts and reduces the risk of ingress of splashed liquids into the circuitry.
• If using a fume hood: power should be supplied via a current limited, PAT tested, external power supply located outside the fume hood, ensuring mains power is not present within the fume hood.
• Cable management systems should be planned to prevent risks of catching.
• All electrical components (roller modules, final take up module, power junction box etc.) should be raised above the level of the work surface/fume hood, this is to reduce the risk of accidental ingress of liquid from spills/splashes.

A note on pump selection/settings:

While specific pump selection is outside the scope of this publication, a few key factors should be considered when selecting a pump for use with this system. Pumps with low pulsation should be selected as pulsation in the flow leads to inconsistencies in the fibre produced. The pump should also be able to pump high viscosity fluids accurately and, at the scale of this spinning system, be able to accurately pump fluids at low flow rates (depending on the spinning dope, spinneret used and process this can range from 0.01 mL/min – 10 mL/min). For basic, first principle research, often a high torque syringe pump strikes the right balance between cost and properties. Micro annular gear pumps offer an excellent continuous flow option that are equipped to deal with high viscosities with low pulsation, and have been validated with this system, but are often expensive.

Authors’ Notes:

Wet spinning, especially at small scale, does involve skill, below are some tips and tricks the author has learnt over 5 + years of wet spinning.

• Degass the spin dope: this can be done either by ultrasound, via vacuum chamber, or even by gentle heating and time to remove all entrapped air bubbles from the spin dope. This will help to reduce inconsistencies in the extruded fibre and prevent fibre breakage.
• Flush pump system with dissolution solvent: whether using a syringe pump or a continuous pump, ensure that all lines and spinnerets have been flushed with dissolution solvent to prevent coagulation of the polymer in the pump/tubing/spinneret.
• Dope viscosity should be such that when pumping through the spinneret enough intrinsic force is generated to ‘push’ the dope away from the spinneret, this helps prevent what is known as ‘die swelling’ in the melt spinning world, or ‘blebbing’ in historical wet spinning texts (a globular mass of solidified polymer at the spinneret tip due to intrinsic attraction of the dope to the spinneret tip). Adding slight tension to the fibre as its being extruded from the spinneret can also help alleviate this, although care has to be taken to not put too much tension on the fibre which can lead to fibre breakage and egress of coagulation dope into the spinneret itself causing blockages.
• Make sure that the spin dope is being pumped uniformly through the spinneret before immersing in the coagulation solvent – this is to help prevent coagulation solvent ingress up into the spinneret.
• Slight tension throughout the system helps in fibres winding efficiently onto the rollers and through the drying system, this can be achieved by running each subsequent roller slightly faster than the previous one (+0.01 m min^−1^ as an example), this can be altered by the user if tension is not high enough.
• When using the roller modules to take fibre out of a bath, it is generally a good idea to elevate the module above the bath, this allows gravity to help ‘seat’ the fibre onto the middle of the roller, otherwise if it is observed that the module/bath are not 100% aligned the fibre can roll off of the module. The height of the module can easily be adjusted using a lab jack until optimal height is found, if the user does not have a lab jack these can be 3D printed (for example: https://www.thingiverse.com/thing:925556) [13]
• When guiding the fibre through the drying system in particular, tension is absolutely essential, this means that an inherent draw ratio will occur during the drying of the fibre, the only way to alleviate this would be to power each individual pulley to remove any intrinsic resistance. Barring this a draw ratio during drying generally improves the tensile properties of the fibre anyway.
• A glass rod is useful when guiding the fibre through the drying system: wrap the fibre multiple times round the end of the glass rod until it locks, then use this glass rod to guide the fibre on a convoluted path through the drying system, due to the fact that the fibre is being moved left to right, and the fibre wrapped around the glass rod is drying, a point of weakness is often observed directly where the fibre starts wrapping round the glass rod, it is therefore advised once in a while to wrap a few more turns onto the rod to replace the portion of fibre at this ‘weak point’. There is probably a better method for drying the fibre, and the author encourages users to develop their own designs and share with the community.
• It is important to remember that only the roller modules and the final take up module are powered and therefore wind the fibre actively. The drying module is un-powered and relies on bearings, this comes along with an inherent resistance as no bearing is perfect. If the fibre being spun is very weak or brittle it may be worth slowing the spinning down and reducing the convolutions taken through the drying module to reduce the friction added into the system while retaining an adequate drying time.
• Table 9 describes common issues, their likely cause and remediation steps to take. This is not an exhaustive list as many of the issues faced by wet spinning are unique to the specific wet spinning process.

**Table 9:** Common spinning issues and causes/resolutions.

Troubleshooting	
Issue	Cause/Resolution
Air bubbles in extruded fibre causing fibre breakage.	Likely cause(s): air present in either the dope or the pump lines. Resolution(s): ensure dope has been thoroughly degassed. Ensure there are no leakages in tubing or pump. Ensure pump has equilibrated by pumping through at least 2x the dead volume of the system before immersing the spinneret.

Fibre is ‘blebbing’ or experiencing die swelling – forming blobs at the end of the spinneret leading to inconsistent fibre profile.	Likely cause(s): intrinsic force insufficient to force fibre away from spinneret and overcome the attractive force between the fibre and the spinneret material. Resolution(s): there are a few ways this can be remediated, mostly focusing on increasing the intrinsic force pushing the fibre away from the spinneret. Increase viscosity of dope while retaining the same pump speed. Increase the pump speed. Reduce the spinneret internal diameter. Ensure the spinneret is clean, with no blockages. Explore different spinneret materials.

Poor fibre coagulation.	Likely cause(s): either the coagulation solvent is non-optimised, or the coagulation time is not long enough. If the fibre is coagulating but showing inconsistencies, this is likely due to either the dope being non-uniform, or the pump having not had enough time to equilibrate. Resolution(s): increase time in coagulation bath, by either using a longer bath or slowing the pump/take up speed. Optimise the coagulation solvent to coagulate the fibre faster. Ensure a uniform dope has been formed, allow at least 2x the dead volume of the system to be pumped, and visually confirm consistent flow before immersing the spinneret.
Fibre sticking to rollers.	Likely cause(s): fibre is insufficiently coagulated, or the force ‘pulling’ the fibre off of the roller is insufficient. Resolution(s): increase coagulation time/optimise coagulation solvent (as above). Raise rollers above the surface of the baths to allow gravity to assist pulling the fibre off the rollers. Set each subsequent roller slightly faster than the previous, this keeps tension on the fibre even as it draws/stretches.
Fibre breakage.	Likely cause(s) could be due to various reasons, the tension in the system could be too high, the fibre could be too thin, the coagulation time/efficacy could be too low, the fibre could have inconsistencies causing localised weak points. Resolution(s):Tension in system too high: slow roller speed down, increase pump speed, use less convolutions in the drying system (you may have to decrease overall system speed to keep the same drying time) . Fibre too thin: increase spinneret internal diameter. Coagulation time/efficacy: see ‘poor fibre coagulation’. Fibre inconsistencies: see ‘poor fibre coagulation’

No fibre being extruded.	Likely cause(s): Spinneret blocked, pump stalled/non-operational. Resolution(s): Ensure pump is operational and has a high enough torque to pump the dope viscosity. If it the pump cannot pump the dope then either reduce dope viscosity (usually by including less polymer) or invest in a new pump. When setting up the system, flush the pump and lines with the dope dissolution solvent, this helps prevent the dope coagulating in the lines. If the spinneret is immersed in the coagulation solvent with no flow, then the spinneret is blocked with coagulated polymer, this can be tested by removing the spinneret and trying to pass solvent through it. If the spinneret is blocked then unblocking can be achieved via sonication in appropriate solvent or via degradation of the polymer via heat, this can be achieved in a muffle furnace or via direct application of heat through an open flame – COSHH forms and risk assessments should be made before attempting this. **Always ensure there is consistent dope flow through the spinneret before immersing in coagulation solvent.**

Fibre sticking to itself on final spool.	Likely cause(s): most likely due to insufficient drying time, meaning the fibre still has coagulation/dissolution solvent present in the polymer matrix. Resolution(s): increase drying time by either slowing the overall speed of the system down, or by increasing the number of convolutions taken by the fibre through the drying system.

Fibre is ‘rolling’ off the rollers, falling off the modules.	Likely cause(s): likely due to insufficient tension in the system or misalignment of the modules. Resolution(s): ensure modules are aligned so that the fibre runs in a straight line from module to module in line with the pulleys. Increase tension through system by running each subsequent module slightly faster than the previous. Elevate roller modules above the baths (start at roughly 20 cm above the surface and adjust accordingly), this allows gravity to help ‘sit’ the fibre into the pulley groove, reducing the potential of the fibre rolling off the module.

## 7 Validation and characterization

**Fig. 31:**
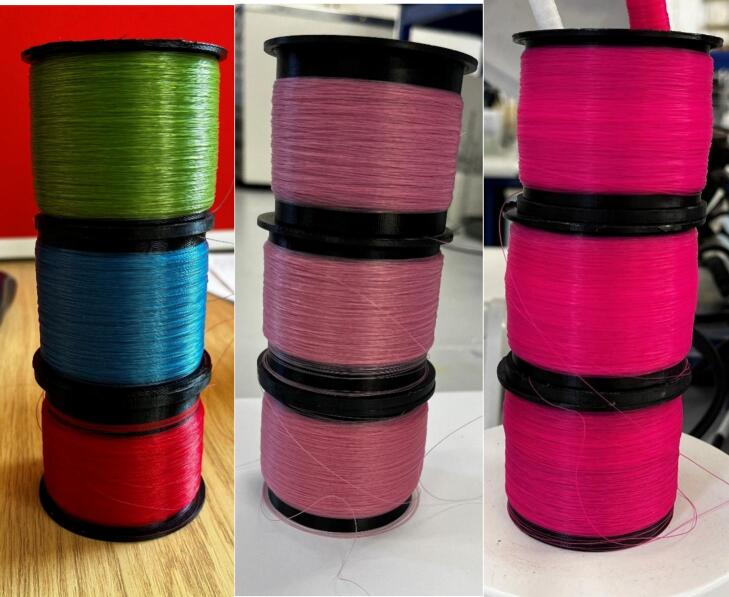
Examples of fibres spun using the system described.

This system has been used for small-scale rapid throughput of wet spinning of various fibres, including casein, alginates and cellulosics (Fig. 31). It has led to previous work on a novel *in-situ* colouration [11] further research is in progress that uses this system.

As an example of the rapid throughput of experimental conditions possible with this system, and using the colouration paper as inspiration, a trial in which 5 different dyes were trialled over the course of one 8 h workday. The entire system was set up, equilibrated and each dye was used to spin approx. 200 m of fibre, the system was cleaned, shut down and packed away successfully within this 8 h period. Only 100 mL of each dye was required, and only 200 g of spin dope was prepared. 200 m of fibre was enough to perform colorimetric analysis, tensile testing, and was even enough for knitting and weaving trials to make swatches.

• Minimum Fibre Uptake (rollers): 0.1 m min^−1^
• Maximum Fibre Uptake (rollers): 2.5 m min^−1^
• Take Up Accuracy (rollers): +/- 0.02 m min^−1^ (below 25 kg·cm torque at minimum speed, below 2.5 kg·cm at max speed)
• Minimum Fibre Uptake (60 mm diameter bobbin): 0.2 m min^−1^
• Maximum Fibre Uptake (60 mm diameter bobbin): 4.5 m min^−1^
• Take Up Accuracy (60 mm diameter bobbin): +/- 0.04 m min^−1^ (below 25 kg·cm torque at minimum speed, below 2.5 kg·cm at max speed)
• Maximum run time without a restart: ∼50 days – this is due to a quirk of the coding: as we are using millis() function to measure the rpm of the motors, and as we are storing it as an unsigned long variable and the Arduino can store values up to 4,294,967,295. This means that once the unsigned long variable exceeds this value it resets to zero, causing the system to incorrectly calculate the rpm as negative causing the Arduino to set the motor power to maximum. There are some elegant ways to code work arounds for this, but the Author deemed that ∼50 days of continuous usage (which is what this equates to) was plenty for a system of this size.

All of these operation parameters are due to the diameter of the rollers/spools and the motors used within the system (motors used have a maximum RPM of 28). Therefore, these values could easily be changed to fit a specific use case by either changing the diameter of the rollers/spools (and editing the relevant line of code as detailed in the code document) or changing the motor for one with either higher or lower RPM (which would work as long as it had the same dimensions as the current used motor, otherwise a redesign of the case would be needed, which again, is not beyond the realm of possibility).

A note on system operational envelope: the pump selection is the main limitation to the operational envelope possible with this system. The pump selection directly limits the dope viscosity range and minimum/maximum dope volume (for example, if a syringe pump is used, the viscosity is limited by the torque of the motor, the maximum dope volume is limited by the size of syringe compatible with the pump, and the minimum dope volume is limited by the dead volume of the pump system. If a continuous pump is used, the maximum dope volume is theoretically infinite, as any size of vessel can be used with a continuous pump. There are no real limits to the spinneret internal diameters, so long as an adequate pump system is chosen, with multi-filament 50 μm spinnerets and wide bore 12G monofilament spinnerets being used effectively. The bath volumes are limited purely by the 3D printed components, with the two baths provided in the design files allowing for ∼50 ml-500 mL, however this can easily be altered by designing and printing/purchasing different size baths.

## Ethics statements

This work involved the development and testing of a low-cost wet spinning system; no human or animal studies were conducted in this work.

## CRediT authorship contribution statement

**Joseph A. Houghton:** Conceptualization, Methodology, Software, Validation, Investigation, Writing – original draft, Writing – review & editing, Funding acquisition. **Richard S. Blackburn:** Supervision, Funding acquisition.

## Declaration of competing interest

The authors declare that they have no known competing financial interests or personal relationships that could have appeared to influence the work reported in this paper.
